# The Accuracy of an Optical White Light Desktop 3D Scanner and Cone Beam CT Scanner Compared to a Multi-Slice CT Scanner to Digitize Anatomical 3D Models: A Pilot Study

**DOI:** 10.3390/cmtr18020027

**Published:** 2025-04-25

**Authors:** Mauranne Lievens, Lisa De Kock, Matthias Ureel, Geert Villeirs, Wim Van Paepegem, Renaat Coopman

**Affiliations:** 1 Department of Oral & Craniomaxillofacial Surgery, Ghent University Hospital, 9000 Ghent, Belgium; mauranne.lievens@ugent.be (M.L.); lisa.dekock@uzgent.be (L.D.K.); matthias.ureel@uzgent.be (M.U.); 2 Department of Medical Imaging, Ghent University Hospital, 9000 Ghent, Belgium; geert.villeirs@uzgent.be; 3 Department of Materials, Textiles and Chemical Engineering, Faculty of Engineering and Architecture, Ghent University, 9000 Ghent, Belgium; wim.vanpaepegem@ugent.be

**Keywords:** digitization, 3D model, medical device regulation, optical scanner, CT scan

## Abstract

Additive manufacturing, in combination with virtual surgery planning, leads to the predictability of complex surgical cases. To guarantee patient safety, three-dimensional (3D) print quality must be ensured and verified. The aim of this study is to compare the accuracy of an optical white-light desktop scanner (OWLDS) and a cone beam CT (CBCT) scanner to that of a multi-slice CT scanner (MSCT) for scanning and digitizing 3D anatomical models. Twenty-two removable parts of a CE-certified anatomical skull, used as a patient-specific surrogate in a clinical workflow, were each scanned by MSCT, CBCT, and OWLDS scanners. The accuracy of the scanning modalities was investigated through a part comparison analysis of the stereolithography (STL) files derived from the different scanning modalities. The high-resolution OWLDS STL files show the smallest overall surface match deviation, at 0.04 mm, compared to the MSCT STL files. The CBCT STL files show an overall deviation of 0.07 mm compared to the MSCT STL files. This difference between the scan modalities increases as the volume of anatomical models decreases. The OWLDS is a safe, cost-effective, user-friendly, and highly accurate scanning modality suitable for accuracy evaluation during the manufacturing process of in-house 3D models. For smaller models, high-resolution optical scans are recommended.

## 1. Introduction

Additive manufacturing (AM), or 3D printing technology, has become an indispensable tool for the craniomaxillofacial (CMF) surgeon to transfer patient-specific virtual surgical planning (VSP) to the operating room in the form of anatomical models, surgical cutting guides, and occlusal splints [1,2,3,4,5,6,7,8,9,10]. Typically, a multi-slice computed tomography (MSCT) scan of the patient is performed first in a VSP workflow. Using medical segmentation software, digital anatomical structures or models are visualized and created by exporting them in a digital Standard Tessellation Language format (STL file). Following segmentation, VSP, based on computer-aided design (CAD), can be performed according to the patient’s needs. Using computer-aided manufacturing (CAM), these STL files can by produced by 3D printing [9,10,11].

The accuracy of the final 3D printed model has an important impact on the patient’s surgical result and is influenced by many factors, e.g., segmentation accuracy, STL design, type of 3D printing, and type of 3D printing material [4,5,6,7,12,13,14]. A necessary step for patient safety is, therefore, to verify, through digitization and comparison analysis, that the shape and volume of the designed STL file and the 3D printed model are equal. Digitization is the process of converting information (e.g., a 3D model) into a digital (i.e., computer-readable) format (e.g., an STL file).

Today, no clear standardized method for digitizing 3D anatomical models exists to verify their accuracy. Some authors have already described the use of a control step in which the 3D-printed models are digitized and surface-matched with the initial virtual STL file [13,15]. Several methods are available to digitize 3D models, including manual measurement, cone-beam CT (CBCT) scans, MSCT scans, and/or an OWLDS [7,8,16,17]. Making measurements manually is subjective and challenging to replicate. Hence, alternatives are preferred for generating measurement data that are consistent and reproducible [7,8].

In a CMF setting, 3D models of the human body are often created using an MSCT scanner. MSCT, using a fan-shaped X-ray beam to capture a series of slices from a continuous spiral motion over the axial plane, is the gold standard for creating detailed 3D images of the human skeleton. MSCT scans are highly accurate and standardized due to the expression of radiodensity in Hounsfield units (HUs) [16,17,18,19].

Secondly, CBCT scanners, an alternative CT technique, use a cone-shaped X-ray beam to capture a 3D image in a single rotation, usually with a much smaller field of view, and are typically used to produce detailed 3D images of a specific bony area of the human body. CBCT scanners are mainly used in dentistry and craniomaxillofacial surgery because of their accessibility, lower cost, and lower radiation exposure [19]. Radiodensity in CBCT is usually expressed in quantitative Gray Values (qGVs) [20].

Thirdly, an OWLDS uses structured light to capture the geometry of an object, which can be used to create a 3D model through reverse engineering. Such scanners are increasingly used in medical settings because of their lower cost, ease of use, accessibility, higher spatial resolution, and non-ionizing radiation imaging. Also, textural information (e.g., color) can be scanned. This additional information has already been described by radiation oncologists, who used it to plan the target volume delineation on the virtual patient model without radio-opaque markers [21].

Limited studies are available on the comparison of imaging modalities to digitize anatomical 3D models. Even though manufacturers often provide information about the accuracy of their products, this information may not be fully applicable in real-world clinical settings. Additionally, many accuracy tests are carried out on objects with symmetrical shapes, but the use of anatomical models may reveal larger inaccuracies in dimension [7,22].

Performing a new MSCT scan to verify the accuracy of every 3D-printed model is excessive and not cost-effective in a clinical setting. Due to the easy accessibility of CBCT and OWLDS scanners in both a hospital and private setting, they seem promising in the accuracy analysis of 3D-printed anatomical models. Limited studies have been carried out comparing these digitizing modalities.

We aim to evaluate the accuracy of STL files created using an OWLDS and CBCT scanner compared to the STL files created using an MSCT scanner (the gold standard).

## 2. Materials and Methods

### 2.1. Digitization of 3D Models

**Figure 1:**
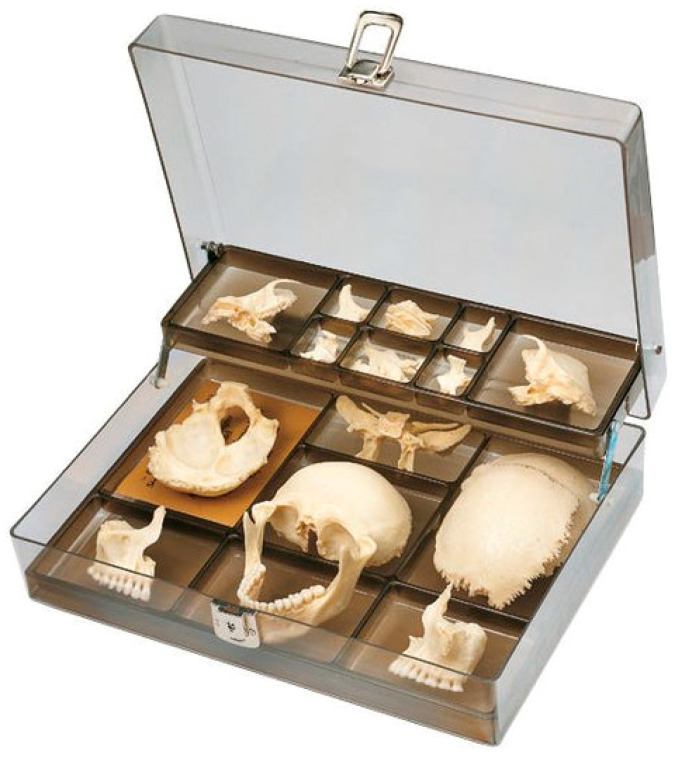
The plastic skull used for this study (SOMSO-Plast^®^ Bauchene QS9/1).

Since this study concerns the accuracy of scanning modalities and to avoid possible bias from the 3D printing procedure, reproducible, CE-certified anatomical models were used. A CE-certified anatomical skull (SOMSO-Plast^®^ Bauchene QS9/1, SOMSO, Coburg, Germany), adequate for educational purposes, was used as a representation or patient-specific surrogate of complex models often used in clinical practice (Figure 1). This skull served as an individual patient model, with different removable anatomical 3D objects (22 in total). Each bone part was individually scanned with an MSCT scanner, CBCT scanner, and OWLDS.

An MSCT using a high-resolution protocol intended for objects and not for patients was performed with the following acquisition settings: system—tube current: 420 milliampere (mA); voltage: 120 kilovolt (kV); 0.6 mm slice thickness; 0.4 mm increment; pitch factor 0.8; rotation time: 1 s (Somatom Definition Flash, Siemens Healthineers, Erlangen, Germany). A small sponge was placed below each object to separate the object from the scanning table.

A CBCT scan of each object was performed (Planmeca; 96 kV; 5.6 mA; scanning time of 12 s.) and the smallest possible field of view was used for each object.

The Digital Imaging and Communications in Medicine (DICOM) images were exported from the MSCT scanner and CBCT scanner and imported into Mimics Innovation Suite (MIS^®^) version 26.0 (Materialise, Leuven, Belgium). The segmentation was performed using the same threshold value for each object (−200 HUs).

**Figure 2:**
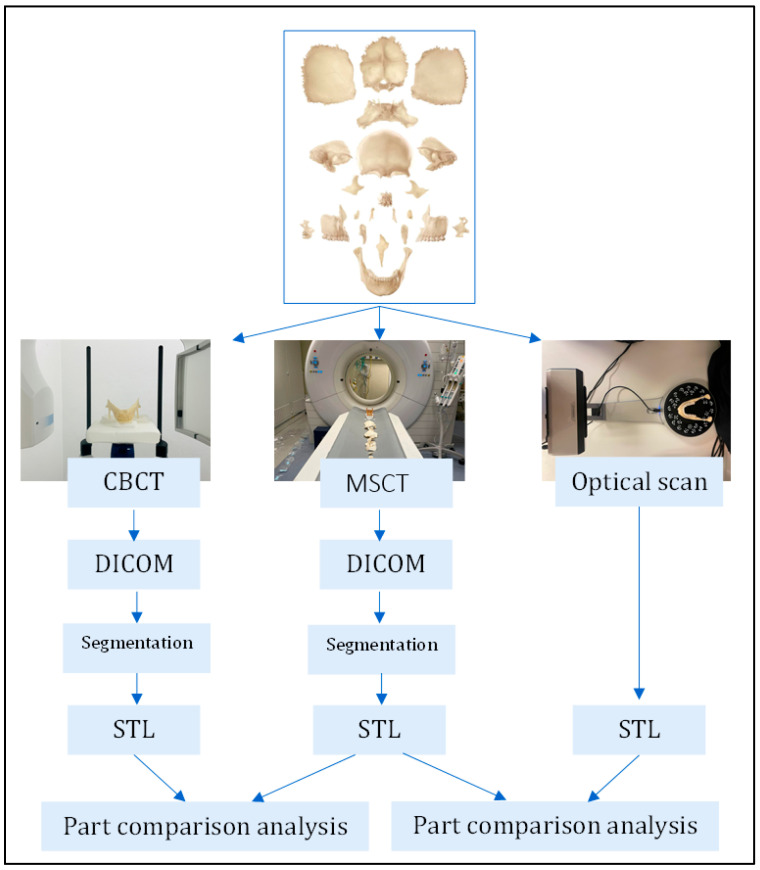
Schematic outline of the study methodology.

An OWLDS (EinScan-SP, SHINING 3D Tech. Co., Ltd., Hangzhou, China) was used to create an optical scan of each object (Figure 2). The OWLDS utilizes white light scanning technology with a single shot accuracy of ≤0.5 mm, a point distance of 0.17–0.2 mm, and a camera resolution of 1.31 megapixels. No additional surface treatment was necessary prior to the scanning procedure. There was constant ambient lighting in the room, and a black background was fixed behind the turntable to enhance contrast. The following specifications were chosen in the Shining 3D Einscan SP software (EXScan S_V3.1.3.0): non-texture scan; high dynamic range (HDR) off; align features; right camera on. Each model was fixed with a removable adhesive putty (3M Scotch, St. Paul, MN, USA) on the turntable and scanned in four different positions: upside, upside down, right side up, and left side up. Eight steps were used for each scan. These four scans were merged into one digital 3D STL model. A non-watertight model was created, and no post-processing was performed. The models were exported as high-resolution STL files.

Additionally, a simplification of the high-resolution STL was performed to match the triangle count of the same object scanned by CT and CBCT. This simplified STL will now be referred to as ‘low-resolution STL’.

### 2.2. Comparison Analysis

For each model, four STL files were created (CT scan, CBCT scan, high-resolution optical scan (HROS), low-resolution optical scan (LROS)). These were imported into MIS^®^ for comparison analysis.

Three comparison analyses were conducted: the high-resolution and low-resolution STL created by the OWLDS compared to the STL created by segmentation of the MSCT scan, and the STL created by segmentation of the CBCT scan compared to the STL created by segmentation of the MSCT scan. These analyses were executed for all 22 individual parts. An STL-file is a triangular representation of a three-dimensional surface geometry. The surface is tessellated or broken down logically into a series of small triangles. Each triangle is described by a perpendicular direction and three points representing the points of the triangle. The surface area is the sum of all the individual triangles. The volume is the amount of space taken up by the STL file.

**Figure 3:**
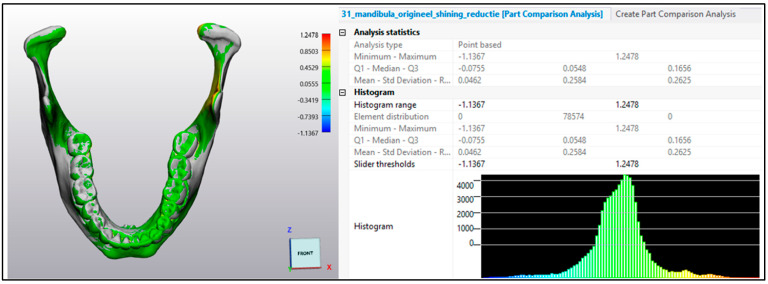
Example of the part comparison analysis tool in Mimics Innovation Suite (MIS^®^) for a comparison analysis of two 3D models of the mandible. A heat map shows the areas of aberrations.

The two models were first roughly aligned through the ‘N-point registration’ tool by using six manually placed control points. The ‘global registration’ tool was performed next to ensure maximum possible superimposition between models. This is a semi-automatic algorithm that decomposes an object into a 3D cloud of voxel points and aligns the positions of all the vertices of the two registered meshes with each other. To assess possible deviations of both meshes from each other, part comparison analysis was conducted based on point cloud comparison. A heat map was created showing the areas of aberrations based on the calculation of the distances between corresponding vertices (Figure 3).

**Figure 4:**
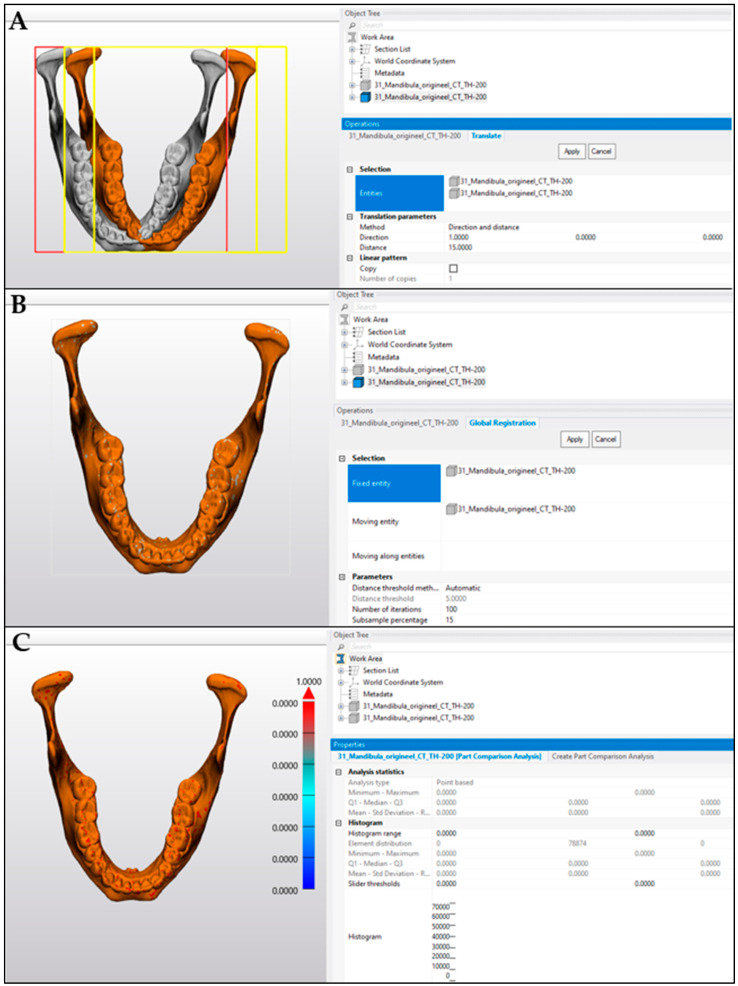
Validation of the part comparison analysis tool in MIS^®^; (**A**) Two identical 3D objects (STL-format) were uploaded in the software and an artificial deviation of one object was created with a translation of 15 mm; (**B**) alignment of the two 3D objects was performed through ‘N-points alignment’ and ‘Global Registration’; (**C**) Part Comparison Analysis shows a mean distance of 0 ± 0.0000 mm.

The validity of the part comparison analysis was evaluated first by aligning two identical 3D objects (the STL obtained by segmentation of the MSCT scan of the mandible bone was used in this case) (Figure 4A). A mean distance was measured at 0 ± 0.0000 mm. By translating one object by 15 mm, an artificial deviation was created. Thereafter, the two 3D objects were again aligned through the ‘N points alignment’ and ‘Global Registration’ tools (Figure 4B). A new part comparison analysis showed a mean distance of 0 ± 0.0000 mm (Figure 4C).

### 2.3. Statistics

The 3D STL models were described based on four parameters: volume, area, number of surface triangles, and cloud points, obtained from MIS^®^. Volume was expressed in cubic centimeters (cm^3^). Three-dimensional anatomical models were ordered by size. Statistically significant differences were calculated using MedCalc (Oostende, Belgium) using a non-parametric Wilcoxon signed ranked test. A *p*-value less than 0.05 was statistically significant and marked with an asterisk in the tables.

## 3. Results

The SOMSO skull consisted of 22 different anatomical parts (Table 1). Of these, six anatomical bone structures were unique (vomer, ethmoid, sphenoid, occipital, frontal bone, and mandibula); the remaining 16 structures were paired in a left and a right side (nasal, lacrimal, concha, palatine, zygomatic, maxilla, temporal, parietal bone).

Two paired bone structures (nasal right and left, lacrimal right and left) had a volume smaller than 1000 cm^3^. Seven bone structures (vomer, concha right and left, palatine right and left, and zygomatic right and left) had an intermediate volume (1000–10,000 cm^3^). The other STL models (ethmoid, maxilla R&L, sphenoid, temporal R&L, occipital, parietal R&L, mandible, and frontal) were larger than 10,000 cm^3^.

Comparing the radiological scanning modalities (MSCT vs. CBCT), no statistically significant differences were found between the STL volumes. When comparing the STL volumes of the OWLDS and MSCT, a statistically significant difference was noticed in volume in the case of the sphenoid bone and the right and left maxilla (*p*-value < 0.05).

Table 2 shows the data for the different models (low resolution versus high resolution) created by the OWLDS. No statistically significant difference was found between the volume and surface area. In the high-resolution models, a significant increase (*p* < 0.0001) was found in the number of triangles and cloud points compared to the low-resolution models, resulting in the 3D models being presented in more detail.

The results of the part comparison analysis (MIS^®^) between the different scan modalities are shown in Table 3. An overall deviation of 0.07 mm, 0.06 mm, and 0.04 mm was found between the 3D models obtained from the MSCT scan compared to the CBCT scan, LROS, and HROS, respectively. This overall deviation (diff mean) was statistically significant between the HROS, the LROS and the CBCT scan.

In general, it is noted that the high-resolution optical scan produces the fewest deviations in the part comparison analysis compared to the MSCT scan. This deviation between the different scan modalities increases as the volume of anatomical shapes decreases. The mean deviation for models smaller than 1000 cm^3^ is 0.08 mm for CBCT and 0.18 mm and 0.12 mm for LROS and HROS, respectively. As the models become larger, this deviation becomes smaller for the high-resolution scan. In general, the deviation between the CBCT scan and the MSCT scan remains the same.

Upon closer examination, outliers in the average deviation were noticed in one anatomical model: the sphenoid bone with 0.23 mm (SD 0.180 mm), 0.19 mm (SD 0.223 mm), and 0.19 mm (SD 0.215 mm) with the CBCT, LROS, and HROS, respectively (marked in bold in Table 3).

## 4. Discussion

The aim of this study was to independently compare different scanning modalities to digitize 3D anatomical objects based on a surgical workflow, and hence to improve and optimize the safety and quality of current clinical procedures.

Today, the most frequently used modality for the digitization of individual anatomical 3D models is an MSCT scan; i.e., the patient undergoes an MSCT scan from which anatomical structures are segmented to perform virtual surgical planning. Therefore, MSCT was set as the gold standard.

As a surrogate for the individual patient, a plastic CE-certified skull was chosen due to its homogeneity and ability to reproduce the current study in other centers.

**Figure 5:**
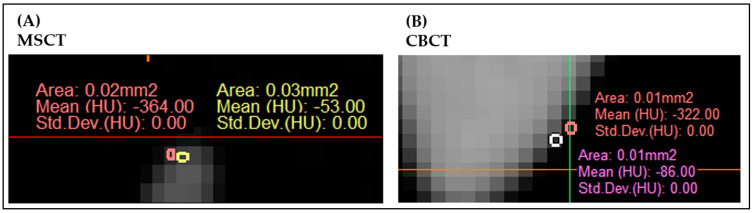
Presentation of voxel values of an MSCT (**A**) and CBCT scanned model (**B**). The average density of each voxel at the outline of the models was measured at −200 HUs in both scanning modalities. At the outline of the model, noise can be noticed.

During MSCT acquisition, a sponge was placed between the plastic bed and the skull bone. This was performed to prevent merging of both objects at the segmentation stage since the plastic skull and the plastic mattress of the scanning table were found to have similar radiodensities. The average radiodensity, expressed in Hounsfield units (HUs) in the MSCT scan, was measured at 115 HUs in the center. When a threshold during segmentation was set at 115 HUs, noise on the surface was noticed, especially in the small models, (Figure 5A). The more noise there was, the more difficult it was to perceive differences in image density. The average density of each voxel at the edges of the models was measured at −200 HUs. This value was chosen as the threshold value to ensure the precise and complete segmentation of each model and to obtain a clear separation from the surrounding air. However, the more noise there was, the more surface detail was lost during segmentation, and the less accurately the foramina could be segmented.

To ensure the highest possible CBCT scan resolution, the smallest field of view was chosen for each object [23]. This field of view was a limitation in some cases. For example, due to the standard positioning of the models and the complex anatomy, it was not possible to completely fit the parietal bone within the largest possible field of view (230 × 100 mm). This problem was solved by stitching two adjacent CBCT scans of the model into one scan series.

The segmentation accuracy of CBCT scans is lower than that of MSCT scans and less standardized [24,25]. Segmentation of MSCT scans is based on HUs, while radiodensity in CBCT scans is expressed in quantitative Gray Values (GVs). GVs are prone to deviation when comparing various CBCT devices, in contrast to the standardized scheme of HUs derived from phantom calibrations in MSCT scans. HUs and GVs show a strong linear relationship, but great variability of GVs can exist on CBCT images due to higher noise levels, more scattered radiation, beam-hardening artifacts, limited field size, and the limitations of currently used reconstruction algorithms [20,26]. According to the software, a threshold of −200 HUs was empirically chosen based on the average density of voxels at the edges of the models, as was applied to MSCT scan segmentation (Figure 5B).

The accuracy of the 3D model created using an OWLDS may be affected by other factors, such as the size and composition of the object, the scanning resolution, the scanning distance, lighting conditions, the reflectivity of the surface, and the post-processing of the 3D scan data [7,14,27,28]. An OWLDS does not rely on image segmentation, as the geometry of the object is directly measured. Most adjustable software algorithms linked to the OWLDS were disabled, and no post-processing was performed with respect to the scan itself (smoothing, hole filling, etc.).

The 3D objects obtained by the OWLDS were found to have, on average, a ten times bigger triangle count compared to the ones obtained by segmentation of CBCT and MSCT scans. This scan was referred to as an HROS. Since the part comparison analysis is based on a point cloud comparison of the 3D objects, a simplification of the STL of the surface scanner was performed to bypass errors and to obtain better comparable data between all digitization modalities. However, by simplifying the 3D object obtained by surface scanning, much detail was lost, and irregularities were visible at the surface. Therefore, this simplified STL was referred to as an LROS.

The accuracy of 3D models can be assessed using 3D deviation analysis or 2D linear measurements [5]. In this study, the analysis was conducted with Mimics Innovation Suite (MIS^®^) (Materialise, Belgium), a CE-certified 3D printing, design, and remeshing software. Other possible software programs (not CE-certified) for surface-based matching of 3D point clouds are Matlab, Cloudcompare, and Meshlab [6,12,29,30,31,32]. Materialise focuses on the applications of 3D software in the medical world and has been used in numerous previous studies on this subject [3,6,7,12,13,33].

Following the example of previous studies that used the part comparison analysis of Materialise, mean difference (MD), standard deviation (SD), and root mean square (RMS) were used for further statistical analysis [7,13,33].

In general, the HROS scans show the smallest deviation compared to the MSCT scans.

**Figure 6:**
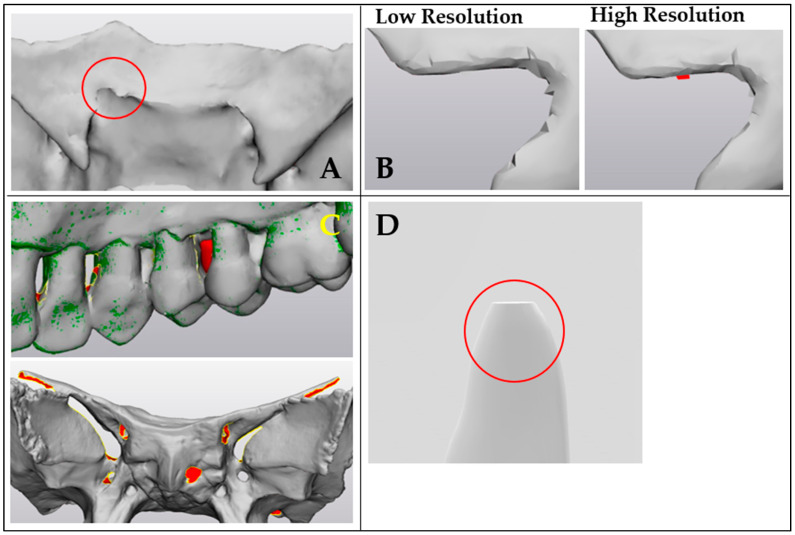
Frequently encountered errors in different scanning modalities. (**A**) Hole in the surface shell, marked with a red circle, around the foramina of the sphenoid bone because of incomplete segmentation of the MSCT scan. (**B**) Close-up view of the surface of the low- and high-resolution optical scans. (**C**) Unscanned undercut areas leaving holes in the mesh, marked in red and/or automatic smoothing algorithms closing the foramina and interdental spaces. (**D**) Influence of squareness of the voxel, marked with a red circle, in the case of small-volume anatomical models.

When comparing the smaller anatomical models, a statistically significant difference was noted between the different modalities in favor of the HROS. We can list two reasons for this. Firstly, surface irregularities are noticed after simplification of the optical scan, resulting in a lower resolution. This has a greater influence on the analysis of smaller volumes (Figure 6B). Secondly, to obtain a 3D model of an MSCT or CBCT scan, segmentation is required, which is based on the indication/thresholding of the voxels, respectively, causing a sharp cut-off after an included slice or voxel (Figure 6D). Segmentation is therefore user-dependent and has a significant influence on the volume of 3D models. In models with smaller volumes, the influence of inaccurate segmentation is even more important.

Additionally, the smaller the object or the higher the complexity, the greater the influence of the noise noted at the surface of MSCT and CBCT scans, resulting in loss of surface detail and less accurate segmentation of foramina (Figure 5 and Figure 6A). This explains why the deviation between CBCT and MSCT is significantly smaller than the deviation between the OWLDS and MSCT in the models with smaller volumes. Although MSCT is considered the gold standard in this study, this observation clearly reveals its biggest disadvantage for smaller models. However, it is imperative to report this due to its importance in daily clinical settings.

Difficulties were seen with the OWLDS in small foramina and undercut areas. This is illustrated by the complex anatomy of the sphenoid bone, explaining the outlier in the part comparison analysis (Figure 6C). This was also seen in the maxilla, where the multiple undercuts of the interdental areas were hard to scan optically (Figure 6C). This explains the significant difference in volume between the OWLDS scans and the MSCT scans for these two models. However, an additional scan can easily be performed from the desired angle where information is missing. Holes in the mesh can be closed by opting for a watertight mesh or by closing the holes manually. This option is available in the software package of the OWLDS. In this study, additional scans and post-processing were avoided to maintain a uniform, standard scanning protocol.

Another OWLDS limitation is the longer processing time when opting for a higher-resolution scan with more steps on the turntable, slower rotation, or adding post-processing options.

A final finding is that models must always be fixed on the turntable. In this study, an adhesive putty (3M Scotch, St. Paul, MN, USA) was used to avoid movement artifacts. A dark background behind the turntable is recommended for optimal contrast.

In recent decades, optical white-light desktop scanners have seen a dramatic increase in the number of clinical applications [28,32,34]. They are relatively cheap, easy to use, and very often mobile or easy to move. In contrast to the OWLDS, CBCT and MSCT scanners are often more expensive, not portable, and always involve ionizing radiation. For the latter reason, the OWLDS requires no special need for infrastructural protection and complies with radiation safety [34].

This study was designed to validate the OWLDS against the MSCT scanner for accuracy analysis of in-house 3D-printed models. A standardized method for digitization of 3D models in healthcare is necessary for the development of a Quality Management System (QMS) in the context of the current EU Medical Device Regulation (MDR, EU 2023/607). Given the clinical importance of this study and follow-up studies, approval from the medical ethics committee was requested and official permission was granted (ONZ-2024-0236), as mentioned above.

The novel contribution of this study is the use of a CE-certified anatomical skull as an in vitro simulation of the clinical setting. No 3D printed models were used because of the potential bias of 3D printing itself. Here, we opted for reproducible CE-certified anatomical models. Accuracy analysis of 3D-printed anatomical models will be the focus in follow-up studies.

Another limitation is the fact that only one type of OWLDS was used in this study. In the future, the same CE-certified anatomical skull can be used as a basic reference to compare the accuracy of different OWLDSs, turning a limitation into an advantage. As for the accuracy analysis of 3D models, the part comparison analysis tool of MIS^®^ remains a user-friendly and validated method.

## 5. Conclusions

This study supports the use of an OWLDS as a scanning modality for digitization and accuracy analysis during the manufacturing process of 3D models, with a recommendation to use a high-precision optical scan with smaller 3D models. For larger 3D models, a CBCT scanner can also be used as an alternative to an MSCT scanner. OWLDSs are user-friendly, relatively cheap, and accessible. In contrast to CBCT and MSCT, there is no need for radioprotection and no segmentation is required to obtain a 3D model, which only increases their applicability in a clinical setting.

## Figures and Tables

**Table 1:** Properties and dimensions of the facial bone models (SOMSO^®^). Volume is expressed in cubic millimeters (mm^3^); surface is expressed in square millimeters (mm^2^). MSCT = gold standard. * *p*-value < 0.05. (Vo = volume; Su = surface; TriA = triangles; Poi = points; R = right; L = left).

Facial Bone	MSCT (Gold Standard)				CBCT (Planmeca)				OWLDS (Low Resolution)			
Part Size (cm^3^)	Vo	Su	TriA	Poi	Vo	Su	TriA	Poi	Vo	Su	TriA	Poi
<1000												
Nasal (R)	**315**	421	8894	4449	**348**	464	6314	3159	**322**	461	8864	4434
Nasal (L)	**287**	406	8724	4364	**249**	419	5210	2607	**321**	476	8770	4385
Lacrimal (R)	**314**	340	7484	3744	**327**	367	4420	2212	**353**	391	7502	3753
Lacrimal (L)	**302**	366	7544	3774	**319**	400	4550	2277	**383**	437	7408	3706
1000–10,000												
Vomer	**1233**	1563	25,748	12,876	**1269**	1662	14,080	7042	**1266**	1687	26,186	13,095
Concha (R)	**1409**	1284	24,080	12,042	**1436**	1346	14,462	7233	**1405**	1364	24,328	12,166
Concha (L)	**1461**	1300	23,874	11,939	**1477**	1362	14,656	7330	**1414**	1373	23,718	11,861
Palatine (R)	**1976**	2031	38,194	19,097	**2062**	2172	29,738	14,865	**2045**	2114	38,484	19,244
Palatine (L)	**1963**	1936	38,724	19,360	**2031**	2055	28,030	14,013	**1994**	2022	38,346	19,175
Zygomatic (R)	**2834**	2479	30,550	15,275	**2905**	2459	27,964	13,984	**2838**	2483	30,832	15,418
Zygomatic (L)	**2804**	2320	30,768	15,386	**3885**	2302	26,392	13,198	**2832**	2324	30,638	15,321
10,000–50,000												
Ethmoid	**12,082**	7361	180,164	90,070	**11,962**	8364	115,156	57,564	**12,664**	5277	180,516	90,952
Maxilla (R)	**20,230**	14,316	125,912	62,954	**20,749**	13,931	133,140	66,580	29,296 *	105,72	128,057	64,378
Maxilla (L)	**19,551**	12,951	125,802	62,905	**20,057**	12,712	129,976	64,988	23,751 *	10,264	125,341	63,284
Sphenoid	**24,161**	18,861	168,822	84,363	**28,346**	19,334	167,758	83,845	32,820 *	16,945	166,433	83,747
Temporal (R)	**23,861**	13,337	129,098	64,549	**24,471**	13,138	126,772	63,384	**24,799**	12,835	129,304	65,005
Temporal (L)	**24,703**	12,679	128,776	64,388	**25,116**	12,493	143,880	71,940	**23,962**	12,200	128,650	64,720
Occipital	**48,940**	27,104	138,542	69,267	**49,742**	26,945	140,198	70,089	**49,586**	27,154	138,736	69,472
Parietal (R)	**44,409**	29,688	120,642	60,323	**39,041**	27,656	121,888	60,932	**44,948**	29,854	119,228	59,664
Parietal (L)	**47,063**	29,643	120,914	60,457	**47,207**	28,581	123,574	61,789	**47,445**	29,744	113,789	56,920
Mandible	**47,173**	20,205	157,732	78,874	**47,757**	19,751	178,322	89,179	**47,928**	18,770	155,921	78,574
>50,000												
Frontal	**71,402**	38,629	209,160	104,580	**74,601**	38,502	92,228	46,112	**72,171**	38,218	218,712	109,645

**Table 2:** Properties and dimensions of the facial bone models (SOMSO^®^). Volume is expressed in cubic millimeters (mm^3^); surface is expressed in square millimeters (mm^2^). * *p*-value < 0.0001. (Vo = volume; Su = surface; TriA = triangles; Poi = points; R = right; L = left).

	Low-Resolution Optical Scan (LROS)				High-Resolution Optical Scan (HROS)			
Part Size (cm^3^)	Vo	Su	TriA	Poi	Vo	Su	TriA	Poi
<1000								
Nasal (R)	322	461	8864	4434	322	460	24,580 *	12,292
Nasal (L)	321	476	8770	4385	322	476	25,042 *	12,521
Lacrimal (R)	353	391	7502	3753	353	390	20,336 *	10,170
Lacrimal (L)	383	437	7408	3706	383	437	23,930 *	11,967
1000–10,000								
Vomer	1266	1687	26,186	13,095	1269	1687	96,958 *	48,481
Concha (R)	1405	1364	24,328	12,166	1405	1364	71,594 *	35,799
Concha (L)	1414	1373	23,718	11,861	1404	1371	65,926 *	32,965
Palatum (R)	2045	2114	38,484	19,244	2046	2114	109,918 *	54,961
Palatum (L)	1994	2022	38,346	19,175	1991	2022	106,576 *	53,288
Zygomatic (R)	2838	2483	30,832	15,418	2839	2482	128,496 *	64,250
Zygomatic (L)	2832	2324	30,638	15,321	2843	2327	119,188 *	59,596
10,000–50,000								
Ethmoid	12,664	5277	180,516	90,952	12,663	5276	278,509 *	139,948
Maxilla (R)	29,296	10,572	128,057	64,378	29,298	10,570	584,510 *	292,633
Maxilla (L)	23,751	10,264	125,341	63,284	23,758	10,263	549,072 *	275,181
Sphenoid	32,820	16,945	166,433	83,747	32,830	16,935	929,369 *	465,259
Temporal (R)	24,799	12,835	129,304	65,005	24,801	12,831	721,532 *	361,144
Temporal (L)	23,962	12,200	128,650	64,720	23,973	12,196	680,356 *	340,627
Occipital	49,586	27,154	138,736	69,472	49,672	27,147	1,543,906 *	772,037
Parietal (R)	44,948	29,854	119,228	59,664	44,943	29,846	1,704,564 *	852,377
Parietal (L)	47,445	29,744	113,789	56,920	47,514	29,742	1,626,060 *	813,102
Mandible	47,928	18,770	155,921	78,574	47,932	18,766	1,046,310 *	523,850
>50,000								
Frontal	72,171	38,218	218,712	109,645	72,168	38,209	2,192,823 *	1,096,742

**Table 3:** Comparison between OWLDS, CBCT scans and MSCT scans. (SD = standard deviation; RMS = root mean square; LROS = low-resolution optical scan; HROS = high-resolution optical scan). * *p*-value < 0.05. (Dev = mean difference; SD = standard deviation; RMS = root mean square; R = right; L = left).

PART COMPARISON		CBCT vs. MSCT			LROS vs. MSCT			HROS vs. MSCT		
Part Size (cm^3^)	Model	MD	SD	RMS	MD	SD	RMS	MD	SD	RMS
<1000	Nasal (R)	0.12	0.131	0.176	0.08	0.160	0.178	0.03	0.145	0.148
	Nasal (L)	0.03	0.164	0.167	0.18	0.247	0.304	0.10	0.180	0.206
	Lacrimal (R)	0.09	0.133	0.159	0.17	0.180	0.247	0.12	0.142	0.185
	Lacrimal (L)	0.09	0.139	0.167	0.29	0.253	0.383	0.22	0.170	0.278
	**Average**	**0.08**	**0.14**	**0.17**	**0.18**	**0.21**	**0.28**	**0.12**	**0.16**	**0.20**
1000–10,000	Vomer	0.09	0.163	0.185	0.08	0.178	0.196	0.03	0.136	0.140
	Concha (R)	0.06	0.144	0.155	0.02	0.154	0.156	0.00	0.130	0.130
	Concha (L)	0.04	0.111	0.118	0.01	0.133	0.134	0.04	0.115	0.120
	Palatum (R)	0.07	0.144	0.159	0.05	0.142	0.149	0.02	0.117	0.120
	Palatum (L)	0.06	0.135	0.146	0.04	0.132	0.138	0.01	0.112	0.113
	Zygomatic (R)	0.03	0.053	0.059	0.01	0.062	0.062	0.00	0.049	0.050
	Zygomatic (L)	0.03	0.055	0.065	0.01	0.142	0.143	0.02	0.051	0.055
	**Average**	**0.05**	**0.11**	**0.13**	**0.03**	**0.13**	**0.14**	**0.02**	**0.10**	**0.10**
10,000–50,000	Ethmoid	0.11	0.384	0.399	0.02	0.157	0.158	0.02	0.139	0.140
	Maxilla (R)	0.04	0.072	0.082	0.02	0.068	0.070	0.02	0.052	0.054
	Maxilla (L)	0.04	0.073	0.084	0.01	0.070	0.070	0.01	0.059	0.060
	Sphenoid	**0.23**	**0.180**	**0.290**	**0.19**	**0.223**	**0.295**	**0.19**	**0.215**	**0.285**
	Temporal (R)	0.03	0.076	0.084	0.01	0.121	0.121	0.01	0.111	0.112
	Temporal (L)	0.03	0.063	0.069	0.00	0.085	0.085	0.00	0.071	0.071
	Occipital	0.02	0.169	0.171	0.01	0.117	0.118	0.01	0.117	0.117
	Parietal (R)	0.17	0.455	0.487	0.03	0.120	0.123	0.02	0.104	0.106
	Parietal (L)	0.02	0.250	0.251	0.00	0.410	0.410	0.01	0.193	0.193
	Mandible	0.04	0.095	0.102	0.05	0.258	0.263	0.00	0.242	0.242
	**Average**	**0.07**	**0.18**	**0.20**	**0.03**	**0.16**	**0.17**	**0.03**	**0.13**	**0.14**
>50,000	Frontal	**0.10**	0.226	0.246	**0.01**	0.103	0.104	**0.01**	0.092	0.093
	**Overall average**	**0.07**	**0.16**	**0.17**	**0.06**	**0.16**	**0.18**	0.04 *	**0.12**	**0.14**

## Data Availability

No new data were created or analyzed in this study. Data sharing is not applicable to this article.

## Abbreviations

The following abbreviations are used in this manuscript:

3D	three-dimensional
AM	Additive Manufacturing
CAD	computer-aided design
CAM	computer-aided manufacturing
CBCT	cone beam CT
CMF	craniomaxillofacial
DICOM	Digital Imaging and Communications in Medicine
HDR	high dynamic range
HROS	High resolution optical scan
HU	Hounsfield unit
L	left
LROS	low-resolution optical scan
MD	mean difference
MDR	Medical Device Regulation
MIS	Mimics Innovation Suite
mA	milliampere
mm	millimeter
mV	millivolt
MSCT	multi-slice CT scanner
OWLDS	optical white-light desktop scanner
Poi	points
QMS	Quality Management System
R	right
RMS	root mean square
SD	standard deviation
STL	stereolithography or Standard Tessellation Language
Su	surface
TriA	triangles
qGV	quantitative Gray Values
Vo	volume
VSP	virtual surgical planning
